# Neonatal outcomes associated with maternal arbovirus infection (dengue, Zika, and chikungunya) among pregnant women with obstetric complications in Brazil: a cross-sectional study

**DOI:** 10.1590/0037-8682-0176-2025

**Published:** 2025-12-15

**Authors:** Gabriela Diniz Militão de Albuquerque, Iracema Jesus Almeida Alves Jacques, Priscila Mayrelle da Silva Castanha, Marilia de Albuquerque Sena, Ana Beatriz Giles Guimarães, Leila Katz, Cynthia Braga, Ulisses Ramos Montarroyos

**Affiliations:** 1 Universidade de Pernambuco. Programa de Pós-Graduação Stricto Sensu em Ciências da Saúde, Recife, PE, Brasil. Universidade de Pernambuco Programa de Pós-Graduação Stricto Sensu em Ciências da Saúde Recife PE Brasil; 2 Instituto Aggeu Magalhães, Fundação Oswaldo Cruz, Recife, PE, Brasil. Instituto Aggeu Magalhães Fundação Oswaldo Cruz Recife PE Brasil; 3 University of Pittsburgh, Department of Infectious Diseases and Microbiology, Pittsburgh, PA, United States of America. University of Pittsburgh Department of Infectious Diseases and Microbiology Pittsburgh PA United States of America; 4 Instituto de Medicina Integral Professor Fernando Figueira, Recife, PE, Brasil. Instituto de Medicina Integral Professor Fernando Figueira Recife PE Brasil

**Keywords:** Pregnancy complications, Zika virus infection, Dengue, Chikungunya fever, Newborn

## Abstract

**Background:**

Arboviruses, including dengue (DENV), Zika (ZIKV), and chikungunya (CHIKV), pose serious threats to neonatal health; however, the consequences of maternal peripartum infections, particularly those that are asymptomatic, remain poorly characterized.

**Methods:**

This hospital-based cross-sectional study investigated the adverse neonatal outcomes associated with maternal arbovirus infection among pregnant women hospitalized for obstetric complications in Northeast Brazil (October 2018 to May 2019). Maternal and neonatal data were collected through interviews and reviewing medical records. Acute/recent maternal infections were confirmed by molecular or serological assays. Associations between maternal infection (categorized as any arbovirus, ZIKV-only, or CHIKV-only) and neonatal outcomes were assessed using Poisson’s regression with robust variance, yielding adjusted prevalence ratios (aPR) and 95% confidence intervals (CIs), controlled for maternal covariates.

**Results:**

Among 806 neonates, 131 (16.3%) were born to arbovirus-infected mothers and had a higher prevalence of prematurity (aPR=1.25, 95% CI: 1.00-1.56) and sepsis (aPR=1.74, 95% CI: 1.07-2.83) compared with those born to non-infected mothers. Neonates of ZIKV-infected mothers had a two-fold higher prevalence of sepsis (aPR=2.02, 95% CI: 1.00-4.11) than neonates of non-infected mothers, and a trend was observed for CHIKV (aPR=1.76, 95% CI: 0.97-3.20).

**Conclusions:**

In this hyperendemic region, a high frequency of newborns were exposed to maternal arbovirus infection during the inter-epidemic period, which was associated with increased premature births and neonatal sepsis. These findings underscore the need for increased clinical vigilance and routine screening in endemic areas, necessitating further longitudinal studies to confirm causality and guide management.

## INTRODUCTION

Arthropod-borne viruses (arboviruses), particularly dengue (DENV), Zika (ZIKV), and chikungunya (CHIKV), represent escalating global health threats, with over 13 million cases of DENV and 500,000 CHIKV reported worldwide in 2024^1^^,^^2^. While ZIKV transmission has remained low following the 2015-2016 epidemic, the persistent risk of resurgence underscores the ongoing threat to susceptible populations, including pregnant women^3^. The massive co-circulation of these viruses imposes a substantial economic burden, with annual costs estimated to have peaked at US$31.3 billion following the emergence of ZIKV and CHIKV^4^.

Approximately 90% of the pregnancies worldwide occur in areas with endemic or epidemic arbovirus transmission, exposing a vast population of pregnant women and their neonates to potential infection^5^. Emerging evidence indicates that arboviral infections in pregnant women during the perinatal period (from the 22nd week of gestation) can significantly increase neonatal morbidity and mortality through various mechanisms, including vertical transmission, placental damage, and activation of the maternal systemic inflammatory response^6^. For instance, maternal DENV infection in the perinatal period is associated with premature birth, low birth weight, and severe neonatal complications resembling sepsis^5^^,^^7^. Maternal CHIKV infection has been linked to severe neonatal encephalopathies and myocarditis^5^^,^^8^. Although the teratogenic effects of first-trimester ZIKV infection, such as congenital Zika syndrome^9^, are well documented, data on the adverse outcomes following maternal ZIKV infection during the perinatal period remain scarce^6^.

A critical challenge in this field is that a high proportion (50-80%) of arbovirus infections in pregnant women are asymptomatic or present with non-specific symptoms^5^^,^^10^. Consequently, studies focusing solely on symptomatic, laboratory-confirmed cases likely underestimate the true burden of adverse neonatal outcomes. Furthermore, in endemic regions such as Northeast Brazil, where DENV, ZIKV, and CHIKV co-circulate, serological cross-reactivity and overlapping clinical presentations hinder accurate clinical diagnosis^11^.

The Northeast region of Brazil has been a hotspot for arbovirus epidemics since the introduction of DENV in the 1980s^12^^,^^13^. It was the site of the first major CHIKV outbreak in 2014 and the epicenter of the ZIKV epidemic in the Americas between 2015 and 2016^14^^,^^15^. Moreover, this region bore the highest burden of microcephaly cases attributed to ZIKV during the 2015-2016 epidemic^16^. This epidemiological background, combined with the persistent co-circulation of all three arboviruses, makes this region a critical setting for this research^12^^,^^13^.

Most studies have focused on the adverse outcomes following symptomatic arboviral infections^17^^,^^18^, leaving a critical gap in our understanding of the impact of asymptomatic infections, which represent the majority of cases. To address this knowledge gap, we conducted a comprehensive investigation of active and recent DENV, ZIKV, and CHIKV infections in a cohort of pregnant women hospitalized for obstetric complications in Pernambuco State, Brazil, a hyperendemic arbovirus region, during the post-epidemic period following major ZIKV and CHIKV outbreaks (2018-2019)^19^. This study design allowed for the capture of a broader spectrum of infections, including asymptomatic infections. Here, we report the neonatal clinical features and adverse outcomes associated with maternal arbovirus infections in this cohort, clarifying the perinatal risks posed by these viruses.

## METHODS

### Study design and population

This hospital-based cross-sectional study included live births and stillbirths from a cohort of pregnant women enrolled at a publicly funded maternity teaching hospital, which is also a regional referral center for high-risk pregnancies in Recife, Northeast Brazil. This facility manages approximately 22,000 emergency obstetric consultations and 5,000 deliveries annually. Additional methodological details have been published elsewhere^19^.

The cohort included 780 pregnant and postpartum women hospitalized for obstetric complications who were consecutively enrolled between October 2018 and May 2019. Eligible participants were ≥ 15 years old, resided in Recife’s Metropolitan Region (Pernambuco state), and had a gestational age of ≥ 27 weeks. Obstetric complications included bleeding, hypertensive disorders (gestational hypertension, preeclampsia, and complete or incomplete HELLP syndrome), metabolic conditions (gestational diabetes), intrauterine infections (chorioamnionitis), placental abnormalities (abruption and oligohydramnios), preterm labor, and premature amniorrhexis. Symptoms of acute arbovirus infection (e.g., fever or rash) were not included. 

### Data collection

Maternal data: Sociodemographic data (age, self-reported skin color, education, family income, smoking, and/or alcohol consumption during pregnancy) and clinical characteristics (parity, prenatal care, and obstetric arbovirus infection symptoms) were collected through structured questionnaire interviews during hospitalization. Paired venous blood samples (admission and 10-day follow-up) were tested for arboviral infections. Of the enrolled women, 33 were discharged before delivery.

Neonatal data: Data on adverse outcomes at birth (Apgar score, gestational age, cephalic perimeter, length, and intrauterine growth restriction [IUGR]), during hospitalization (fever, respiratory distress syndrome, pathological jaundice, sepsis, and hospitalization in the intensive care unit), and medical interventions (resuscitation maneuver, ventilatory and nutritional support, antibiotic therapy, and intravenous fluid therapy) were extracted from medical records using standardized methods. Discharge summaries provided by the parents were reviewed for births that occurred at another maternity hospital. All neonatal outcomes were reviewed by the research team according to the case definitions in international guidelines: pathological jaundice^20^, IUGR^21^, microcephaly^21^, respiratory distress syndrome^22^^,^^23^, and neonatal sepsis^24^.

### Laboratory diagnosis of arbovirus infections

Viral RNA was extracted using the ReliaPrep^TM^ Viral TNA Miniprep System (Promega Corporation, Madison, WI, USA) following the manufacturer's protocol. Virus-specific RNA (DENV, ZIKV, or CHIKV) was detected in a single reaction using multiplex real-time quantitative reverse transcription PCR (RT-qPCR)^25^. Positive RT‒qPCR results using the multiplex assay were further confirmed by a single virus-specific RT‒qPCR.

DENV and ZIKV IgM antibodies were detected using an in-house enzyme-linked immunosorbent assay (ELISA) following Centers for Disease Control (CDC) protocols^26^. CHIKV IgM antibodies were detected using a commercial ELISA kit (Euroimmun, Lübeck, SH, Germany) following the manufacturer's protocol.

A virus-specific plaque reduction neutralization test (PRNT) was performed on samples with positive or inconclusive serological results^27^. The complete laboratory methodology has been previously published by Jacques et al.^19^.

### Arbovirus infection definitions

Active infection: Active infection was defined as RNA detection of any arbovirus (DENV, ZIKV, or CHIKV) in the first sample, IgM/IgG seroconversion, or a ≥ 4-fold increase in virus-specific neutralizing antibodies in paired samples (PRNTs).

Recent infection: Recent infection was defined as virus-specific IgM detection in the first or paired samples.

### Data analysis

Data were managed using REDCap (FIOCRUZ, Brazil). Statistical analyses were conducted using Stata 17 software (StataCorp., College Station, TX, USA). Initially, we performed a descriptive analysis to characterize the study population, with the distributions of clinical and neonatal outcomes (at birth and during hospitalization) compared to the maternal arbovirus infection status (uninfected, infected with any arbovirus, ZIKV-only, or CHIKV-only). Differences were assessed using Pearson’s chi-square or Fisher’s exact test for categorical variables and Student’s t-test or the Kruskal-Wallis test for continuous variables, with a statistical significance threshold of p ≤ 0.05.

The association between each neonatal outcome and primary exposure (infection with any arbovirus, ZIKV only, or CHIKV only) was assessed using Poisson’s regression models with robust variance, which directly estimate prevalence ratios (PRs) and 95% Confidence Intervals (CI). All models were adjusted for a set of maternal covariates that were considered potential confounders: hypertensive disorders, diabetes, other comorbidities, number of prenatal care visits, self-reported race/skin color, alcohol use, and smoking during pregnancy. Although some binary outcomes had a prevalence of < 10%, Poisson’s regression was retained for all analyses for consistency and because a comparison with logistic regression models showed that the adjusted PRs (aPRs) and adjusted odds ratios (aORs) were extremely similar, indicating minimal overestimation of the association.

In the categorical explanatory variable, 2.4-2.7% of data were missing, which were handled using multiple imputations using the chained equations (MICE) method. Twenty imputed datasets were generated, and the imputation model included all variables from the final model, including the outcome variables, to support the assumption that the data were missing at random. The results from the imputed datasets were combined according to Rubin’s role^28^. 

### Ethical approval

The study protocol was approved by the Ethics Committee of the Aggeu Magalhaes Institute, Oswaldo Cruz Foundation (CAEE: 73121517.0.0000.5190), and Instituto de Medicina Integral Prof. Fernando Figueira-IMIP (CAEE: 73121517.0.3001.5201). Written informed consent was obtained from the participants or their guardians (aged <18 years). The test results were disclosed, and the neonates of positive mothers received follow-up care.

## RESULTS

**FIGURE 1:**
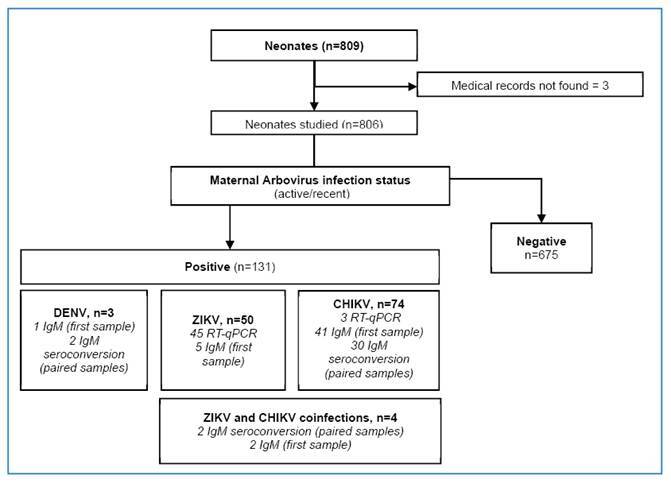
Flowchart of the study population.

This study included 780 pregnant women, with 798 live births and 11 stillbirths. After excluding three neonates with missing records, the final study sample comprised 806 neonates (Figure 1). Of these, 58 (7.2%) were twins, 190 (23.9%) had low birth weight, 244 (30.7%) were preterm, and 651 (85.9%) had adverse outcomes at birth (Figure 1, Table 1). Additionally, 548 (68.0%) were born to mothers with hypertension and 177 (22.0%) to mothers with gestational diabetes.

Among the 131 neonates (16.3%) born to mothers with arbovirus infections, 50 were from ZIKV-infected mothers, 74 were from CHIKV-infected mothers, 4 were from ZIKV/CHIKV-coinfected mothers, and 3 were from DENV-infected mothers. Most neonates (n= 82; 62.6%) were born to mothers with active infections at delivery (Figure 1 and Table 1).

**TABLE 1:** Main characteristics of the study population.

Characteristics	n= 806^a^
**At birth**	
Neonatal outcome, n (%)	
Stillbirth	11 (1.4)
Born alive	795 (98.6)
**Type of birth, n (%)**	
Transvaginal	344 (44.7)
Cesarean	425 (55.3)
**Twin, n (%)**	
Yes	58 (7.2)
No	748 (92.8)
**Birthweight (grams), n (%)**	
<2,500 (low birth weight)	190 (23.9)
≥2,500 e <4,000 (eutrophic)	554 (69.7)
≥4,000 (macrosomic)	51 (6.4)
**Sex, n (%)**	
Male	409 (51.5)
Female	385 (48.5)
**Gestational age, n (%)**	
Preterm (<37 weeks)	244 (30.7)
Term (≥37 and <42 weeks)	547 (68.8)
Post-term (≥42 weeks)	4 (0.5)
**Neonatal complication** ^b^ **, n (%)**	
Yes	651 (85.9)
No	107 (14.1)
**ICU admission, n (%)**	
Yes	74 (9.3)
No	724 (90.7)
Duration of hospitalization (days), median (range)	3 (1 - 58)
**Maternal characteristics**	
**Arboviral infection status (current/recent), n (%)**	
Uninfected	675 (83.7)
Zika	50 (6.2)
Chikungunya	74 (9.2)
Dengue	3 (0.4)
ZIKV/CHIKV coinfection	4 (0.5)
**Arbovirus infection status, n (%)**	
Active (current)^c^	82 (62.6)
Recent^d^	49 (37.4)
**Type of obstetric complication** ^e^ **, n (%)**	
Hypertensive Disorders	548 (68.0)
Gestational diabetes	177 (22.0)
Others	111 (13.8)

a Numbers may vary owing to missing values. ^b^Hypothermia; resuscitation maneuver; respiratory distress; neonatal hypoxia; traumatism. ^c^RT-qPCR positivity in the first sample and/or IgM or IgG seroconversion (PRNT). ^d^RT-qPCR negative and IgM-positive in the first and paired samples. ^e^Gestational hypertension, preeclampsia or eclampsia, HELLP syndrome; bleeding, oligohydramnios, chorioamnionitis, placental abruption, premature amniorrhexis. More than one event may have occurred for each participant.

Table 2 shows the frequency of clinical features of the neonates according to maternal arboviral infection status. Neonates of DENV-infected mothers (n=3) were not analyzed as a separate category because of their low frequency in the study population. Compared with those born to uninfected mothers, neonates born to ZIKV-infected mothers had significantly shorter birth lengths (46.5 cm vs. 48.0 cm; p = 0.01). No other significant differences in the clinical features at birth were observed. During hospitalization, neonates born to arbovirus-infected mothers (any arbovirus) had a higher frequency of adverse outcomes than those born to uninfected mothers (56.1% vs. 46.6%, p = 0.04).

**TABLE 2:** Clinical features, interventions, and birth outcomes of neonates born to women with obstetric complications according to maternal arboviral infection status in a maternity hospital in Northeast Brazil, 2018 - 2019.

Neonatal clinical characteristics	Maternal arbovirus infection status						
	Uninfected N=675	Any arbovirus N= 131	*p-value*	ZIKV^e^ (n= 50)	*p-value*	CHIKV^f^ N= 74	*p-value*
**At birth**							
1-minute Apgar score, median (IQR)^g^	9 (8 - 9)	9 (8 - 9)	0.63^a^	8 (8 - 9)	0.42^a^	9 (8 - 9)	0.87^a^
5-minute Apgar score, median (IQR)^g^	9 (9 - 10)	9 (9 - 10)	0.12^a^	9 (9 - 10)	0.09^a^	9 (9 - 10)	0.43^a^
Prematurity (<37 gestational weeks), n (%)	197 (29.2)	49 (37.4)	0.06^b^	19 (38.0)	0.18^b^	26 (35.1)	0.28^b^
Microcephaly (cephalic perimeter < 2DP), n (%)	16 (2.4)	2 (1.5)	0.75^c^	1 (2.0)	1.00^c^	1 (1.4)	1.00^c^
Length at birth in cm, median (IQR)^g^	48 (45.5-50)	47,1 (44.5-49.2)	0.08^a^	**46.5 (44 - 49)**	**0.01** ^a^	47.5 (44.7-49.5)	0.46^a^
Intrauterine Growth Restriction (IUGR), n (%)	74 (11.0)	18 (13.7)	0.36^b^	7 (14.0)	0.51^b^	10 (13.5)	0.50^b^
**During hospitalization**							
Any neonatal adverse outcome, n (%)	314 (46.6)	**73 (56.1)**	**0.04** ^b^	29 (59.2)	0.08^b^	39 (52.7)	0.31^b^
Fever, n (%)	18 (2.8)	7 (5.7)	0.10^b^	4 (8.5)	0.06^c^	3 (4.3)	0.45^c^
Respiratory distress syndrome, n (%)	64 (9.5)	15 (11.5)	0.51^b^	7 (14.3)	0.27^b^	7 (9.5)	0.99^b^
Pathological jaundice, n (%)	88 (13.1)	21 (16.1)	0.34^b^	11 (22.4)	0.06^b^	10 (13.5)	0.91^b^
Sepsis, n (%)	55 (8.2)	**19 (14.6)**	**0.02** ^b^	**8 (16.3)**	**0.05** ^b^	**11 (14.9)**	**0.05** ^b^
Hospitalization in Intensive Care Unit, n (%)	61 (9.1)	13 (10.2)	0.70^b^	6 (12.5)	0.43^b^	7 (9.6)	0.89^b^
**Medical interventions**							
Resuscitation maneuver, n (%)	157 (24.7)	35 (28.5)	0.38^b^	16 (34.0)	0.15^b^	18 (25.7)	0.85^b^
Ventilatory support, n (%)	97 (15.3)	24 (19.7)	0.23^b^	12 (25.5)	0.06^b^	11 (15.9)	0.89^b^
Nutritional support, n (%)	120 (19.0)	32 (26.0)	0.07^b^	13 (27.6)	0.14^b^	17 (24.3)	0.28^b^
Antibiotic therapy, n (%)	59 (9.3)	18 (14.6)	0.07^b^	8 (17.0)	0.08^b^	10 (14.3)	0.18^b^
Intravenous Fluid Therapy^h^, n (%)	52 (8.3)	13 (10.6)	0.41^b^	6 (12.7)	0.29^b^	7 (10.0)	0.63^b^
**Neonatal adverse outcome**							
Death, n (%)	20 (3.0)	5 (3.8)	0.58^c^	3 (6.0)	0.20^c^	1 (1.4)	0.71^c^

a Kruskal-Wallis; ^b^Qui-square; ^c^Pearson chi-square; ^d^dengue, Zika and/or chikungunya virus; ^e^Zika virus; ^f^Chikungunya virus; ^g^Interquartile range, ^h^electrolytes, blood, and blood products.

Neonatal sepsis occurred more frequently in neonates born to arbovirus-infected mothers than in those born to uninfected mothers (14.6% vs. 8.2%, p = 0.02), ZIKV infections (16.3% vs. 8.2%; P =0.05), or CHIKV infections (14.9% vs. 8.2%, p=0.05). Maternal arboviral infection was not associated with fever, respiratory distress syndrome, pathological jaundice, or other medical interventions (all p > 0.05).

Table 3 shows the results of the adjusted Poisson regression analysis of the association between maternal arbovirus infection and neonatal outcomes after controlling for potential maternal confounders. Compared with neonates from uninfected mothers, those born to mothers infected with any arbovirus had a 25% higher prevalence of prematurity (aPR=1.25; 95% CI: 1.00-1.56; p=0.041) and a 74% higher prevalence of sepsis (aPR=1.74; 95% CI: 1.07-2.83; p=0.026). 

**TABLE 3:** Adjusted prevalence ratio (aPR) of the association of clinical features, medical interventions, and neonatal outcomes with maternal arbovirus infection in a maternity hospital in Northeast Brazil, 2018 - 2019.

Clinical characteristics	Maternal arbovirus infection					
	Any arbovirus^b^		ZIKV^c^		CHIKV^d^	
	aPR^a^ (95%CI)	p-value	aPR^a^ (95%CI)	p-value	aPR^a^ (95%CI)	p-value
**At birth**						
Prematurity (gestational age< 37 weeks)	**1.25 (1.00 - 1.56)**	**0.041**	1.27 (0.91 - 1.79)	0.162	1.20 (0.91 - 1.60)	0.195
Microcephaly (cephalic perimeter < 2DP)	0.58 (0.13 - 2.90)	0.472	0.80 (0.11 - 5.70)	0.826	0.49 (0.37 - 3.66)	0.504
Intrauterine Growth Restriction (IUGR)	1.20 (0.75 - 1.92)	0.455	1.36 (0.65 - 2.83)	0.419	1.10 (0.61 - 1.98)	0.746
**During hospitalization**						
Any neonatal complication	**1.17 (0.99 - 1.37)**	**0.058**	**1.24 (0.97 - 1.58)**	**0.080**	1.10 (0.89 - 1.36)	0.375
Fever	1.91 (0.84 - 4.36)	0.125	**2.77 (0.95 - 8.11)**	**0.063**	1.56 (0.48 - 4.99)	0.461
Respiratory distress syndrome	1.21 (0.71 - 2.04)	0.491	1.52 (0.74 - 3.14)	0.254	0.99 (0.47 - 2.08)	0.982
Pathological jaundice	1.20 (0.78 - 1.84)	0.408	**1.65 (0.95 - 2.87)**	**0.073**	1.03 (0.57 - 1.85)	0.932
Sepsis	**1.74 (1.07 - 2.83)**	**0.026**	**2.02 (1.00 - 4.11)**	**0.053**	**1.76 (0.97 - 3.20)**	**0.063**
Hospitalization in Intensive Care Unit	1.07 (0.60 - 1.89)	0.817	1.40 (0.63 - 3.11)	0.412	0.99 (0.46 - 2.11)	0.974
**Medical interventions**						
Resuscitation maneuvers	1.14 (0.84 - 1.56)	0.403	1.35 (0.89 - 2.10)	0.159	1.03 (0.68 - 1.58)	0.861
Ventilatory support	1.25 (0.84 - 1.86)	0.277	**1.64 (0.98 - 2.75)**	0.061	1.02 (0.58 - 1.79)	0.956
Nutritional support	**1.35 (0.96 - 1.89)**	**0.082**	1.44 (0.89 - 2.35)	0.142	1.27 (0.81 - 1.97)	0.293
Antibiotic therapy	**1.54 (0.95 - 2.50)**	**0.079**	**1.86 (0.95 - 3.64)**	**0.069**	1.50 (0.80 - 2.75)	0.202
Intravenous Fluid Therapy^e^	1.22 (0.68 - 2.20)	0.497	1.60 (0.70 - 3.48)	0.280	1.11 (0.53 - 2.37)	0.772
**Neonatal adverse outcome**						
Death	1.37 (0.53 - 3.53)	0.513	2.19 (0.66 - 7.31)	0.203	0.53 (0.07 - 3.97)	0.533

a Adjusted for the following maternal variables: type of obstetric complication, smoking and/or alcohol use during pregnancy, skin color, and number of prenatal consultations. ^b^dengue, Zika, chikungunya virus, ^c^Zika virus, ^d^chikungunya virus, ^e^electrolytes, blood, and blood products.

Compared with neonates of uninfected mothers, neonates born to ZIKV-infected mothers had a two-fold higher prevalence of sepsis (aPR=2.02; 95% CI: 1.00-4.11; p=0.053), a finding that approached statistical significance. A trend toward a higher prevalence of fever (aPR=2.77; p=0.063) and pathological jaundice (aPR=1.65; p=0.073) was also observed in this group. Furthermore, the neonates of ZIKV-infected mothers had a higher prevalence of ventilatory support (aPR=1.64; p=0.061) and antibiotic therapy (aPR=1.86; p=0.069), suggesting greater clinical severity. For CHIKV infection, a borderline association with sepsis was observed (aPR=1.76; 95% CI: 0.97-3.20; p=0.063).

When the arboviruses were pooled, maternal infection was associated with a higher prevalence of neonates requiring nutritional support (aPR=1.35; p=0.082) and antibiotic therapy (aPR=1.54; p=0.079). No significant association was found between maternal arbovirus infection and microcephaly or IUGR.

## DISCUSSION

Our study provides important insights into the spectrum of neonatal outcomes associated with maternal arbovirus infections in a hyperendemic region of Northeast Brazil. The high proportion of neonates born to arbovirus-infected mothers (16.3%) during the interepidemic period demonstrates the persistent risk posed by these pathogens. These findings align with the growing global body of evidence on the perinatal burden of *Aedes*-transmitted arboviruses. For instance, studies from other endemic regions have similarly reported significant associations between maternal arbovirus infection and adverse outcomes, such as preterm birth and low birth weight, highlighting this issue as a widespread public health challenge beyond the Americas^5^^,^^7^^,^^29^.

Our analysis, which directly estimated PRs, found that neonates born to mothers infected with any arbovirus had a significantly higher prevalence of premature birth (aPR=1.25) and sepsis (aPR=1.74). These findings emphasize the critical importance of routine arbovirus screening for high-risk pregnancies in these regions because the majority of infected mothers are asymptomatic^19^.

Among the ZIKV-infected mothers, nearly all infections were confirmed by RT-PCR, indicating viremia during the perinatal period. In contrast, approximately 50% of the active maternal CHIKV infections were confirmed by seroconversion, with only one case being CHIKV-viremic. Compared to the predominantly serologically confirmed CHIKV infections, the predominance of RT-qPCR-confirmed ZIKV infections suggests that different transmission dynamics may influence neonatal outcomes. Evidence indicates that the typical neonatal symptoms of CHIKV infection are rare or even absent when maternal infection occurs during the prepartum or peripartum period^17^. Thus, the absence of symptomatic CHIKV in our study population may be attributable to the low frequency of maternal CHIKV viremia at delivery. We speculate that the adverse neonatal outcomes associated with maternal CHIKV infection observed in our study likely stem from indirect viral effects such as maternal inflammatory responses or placental damage^6^.

Although congenital Zika syndrome is well documented^9^, few studies have addressed the effects of peripartum ZIKV infection^27^^,^^30^. Regression analysis indicated that neonates born to ZIKV-infected mothers (nearly all with peripartum viremia) had a notably higher prevalence of sepsis (aPR=2.02), with a trend toward a higher prevalence of fever and requirement for ventilatory support and antibiotic therapy. These results, although borderline significant for some outcomes, suggest a trend toward more severe neonatal morbidity in peripartum ZIKV-exposed neonates than in those exposed to CHIKV in our study population.

Surprisingly, we found no association between maternal ZIKV infection and microcephaly, prematurity, or IUGR in neonates despite well-established evidence linking these outcomes to ZIKV infection^9^^,^^31^. Similar null findings were reported by Clemente et al.^32^ in their study of pregnant women with obstetric complications. This discrepancy may reflect the unique clinical profile of our study population, which exclusively comprised neonates of mothers with obstetric complications. Additionally, all maternal infections in our study were detected in the third trimester, a period when the teratogenic potential of the virus is substantially lower than that during the first or second trimesters^31^^,^^33^.

Regarding clinical outcomes during hospitalization, neonates born to arbovirus-infected mothers showed a higher prevalence of neonatal complications, particularly sepsis, than those born to uninfected mothers, although no significant differences were observed in other specific clinical features. These findings are consistent with those of previous studies on neonatal outcomes following maternal arboviral infection. For example, Clemente et al.^32^ reported a strong association between ZIKV infection and congenital microcephaly in high-risk pregnancies in southeastern Brazil but no association with prematurity, low birth weight, small size for gestational age, or fetal death. Similarly, Foeller et al.^34^ and Basurko et al.^35^ reported no significant differences in most neonatal outcomes between CHIKV-infected and uninfected mothers in studies conducted in Grenada and French Guiana, respectively. These consistent results highlight the challenge of distinguishing neonatal outcomes specifically attributable to maternal arbovirus infection from those caused by other underlying factors and highlight the importance of laboratory arbovirus screening to improve diagnostic accuracy.

Consistent with previous findings^7^, our analysis revealed that neonates born to mothers infected with any of the arboviruses had a significantly higher prevalence of premature births (aPR=1.25) than those born to uninfected mothers. This association is biologically plausible given the evidence of virus-induced placental dysfunction, which contributes to adverse perinatal outcomes, including fetal loss, preterm birth, and low birth weight, in mothers with DENV infections^5^. Therefore, the possible pathophysiological mechanisms may involve both direct viral effects on placental tissue and systemic inflammatory responses, with elevated levels of IL-6, TNF-α, and prostaglandin E2 being particularly implicated in arbovirus-associated preterm birth^18^.

Regression analysis demonstrated that neonates born to mothers infected with arboviruses had a 74% higher prevalence of sepsis than those born to uninfected mothers. A similar elevated prevalence was observed for neonates born to ZIKV-infected mothers (aPR=2.02), and a similar trend was observed for those born to CHIKV-infected mothers, although this association was borderline and statistically significant. These findings align with the literature documenting the associations between DENV, ZIKV, and CHIKV infections during pregnancy and neonatal sepsis-like syndrome^6^^,^^17^^,^^36^. The pathogenesis of this outcome may involve direct viral infection of placental cells (e.g., by CHIKV/ZIKV), which triggers cytokine release and complement system activation, ultimately leading to placental inflammation and vascular damage^5^^,^^37^. Additionally, fetal inflammatory response syndrome may develop when maternal cytokines cross the compromised placental barrier, representing another key mechanism underlying neonatal sepsis-like presentations^38^^,^^39^.

A key strength of this study is its cross-sectional design nested within a well-characterized population of pregnant women recruited using standardized inclusion criteria at a tertiary referral hospital. This design allowed the inclusion of participants regardless of their arbovirus symptom status, thereby reducing selection bias. Furthermore, all infections were laboratory-confirmed, enabling the precise ascertainment of both symptomatic and asymptomatic infections.

This study has some limitations. First, the use of secondary data from medical records may introduce information bias owing to incomplete or inaccurate records. However, this risk was mitigated by the study being conducted in a tertiary teaching hospital where experienced clinicians adhered to standardized international protocols for patient care and record-keeping. Second, the statistical power was limited for the analysis of rare outcomes and specific viral subgroups. In particular, the low number of DENV infections (n=3) hindered meaningful assessment of their individual effects. Consequently, our analyses may have been underpowered to detect significant associations with less common outcomes such as microcephaly, increasing the possibility of Type II (beta) errors. Finally, as this was a single-center investigation, the generalizability of our findings to populations with healthcare facilities having different infrastructures or arbovirus transmission dynamics may be limited. These limitations highlight the need for future multicenter studies with larger sample sizes to better characterize rare neonatal outcomes; investigate virus-specific effects, particularly DENV; and validate our findings in diverse epidemiological contexts.

In summary, this study found a high prevalence of neonates exposed to maternal arbovirus infections during the perinatal period in a hyperendemic region of Brazil, even during the inter-epidemic period. Although the cross-sectional design and sample size limitations precluded definitive causal inference, our findings contribute to the understanding of the potential impact of these infections on neonatal health. 

These results suggest that maternal arbovirus infection, particularly in the perinatal period, may be associated with an increased prevalence of adverse outcomes, such as prematurity and sepsis. These observations highlight key considerations for clinical practice and public health: the importance of enhanced surveillance and routine screening in high-risk pregnancies, reinforcement of preventive measures for pregnant women in endemic regions, and development of standardized approaches for diagnosing perinatal arbovirus infections. Finally, these findings underscore the need for evidence-based protocols to manage potentially exposed neonates and warrant further longitudinal studies to establish causality and characterize the long-term consequences of perinatal arbovirus exposure.

## Data Availability

Data-available-upon-request.
